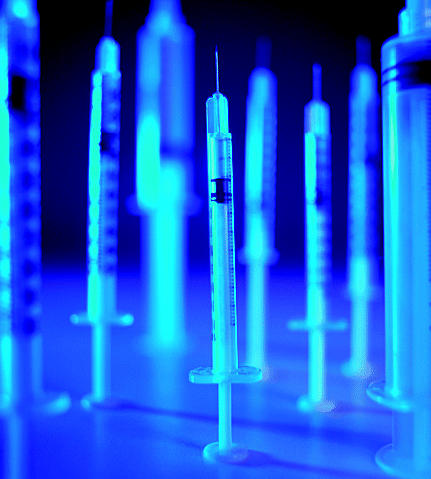# The Beat

**Published:** 2004-10

**Authors:** Erin E. Dooley

## e-Hospitals for Better Care

In July 2004 DHHS secretary Tommy Thompson announced a 10-year plan to improve the delivery of health care across the nation using electronic health records. The plan—initiated by President Bush in April—calls for continuously updated and accessible electronic health records, which can give physicians life-saving information in real time. A recent Institute of Medicine report found that implementation of electronic medical record systems could reduce the tens of thousands of deaths and injuries caused by medical mistakes each year. Bush has called for electronic medical records for most Americans within 10 years. A panel appointed by Thompson should provide a full cost–benefit analysis of health information technology this fall.

## WHO Launches Children’s Health Resources

The World Health Organization has unveiled several new resources on children’s environmental health. An “e-library” CD-ROM contains more than 100 documents concerning children’s environmental health. *Children’s Health and the Environment: A Global Perspective* is a reference manual for health care providers and policy makers that includes case studies of environmental illnesses, tips on taking pediatric environmental histories, and ways people can take action to improve children’s health. And the *Atlas of Children’s Environmental Health and the Environment* is a compendium of facts on environmental hazards to children. These resources are intended to underscore for stakeholders how greatly children are impacted by the environment. It is estimated that over 40% of the environment-related disease burden falls on children under the age of 5, with over 3 million children dying from environmental causes each year.

## Tragic Trinkets

The Consumer Product Safety Commission issued a record-breaking recall in July 2004 when it announced that 150 million toy bracelets, rings, and necklaces should be removed from the market or discarded by parents because they contain high levels of lead. The vending machine trinkets had already been recalled twice before during the preceding year, but the problem persisted. Tests have found that some of the items, which are made in India, contain as much as 69% lead by weight. The four importers of the jewelry have agreed to the recall, while the commission has posted photos of the items on the website **http://www.toyjewelryrecall.com**.

## Preservation Masterpiece

Ozone-depleting methyl bromide was once the fumigant of choice for protecting museum artifacts against insects and mold. But with the chemical’s scheduled phase-out in January 2005, an alternative is gaining ground in museums around the world. The new method uses commercially available oxygen absorbers and airtight bags to kill pests by depriving them of oxygen. The technology was first developed by NASA to prevent rusting, and was first used for artifact preservation in the early 1990s in two California museums. Today it is being used in North America, Australia, Europe, Latin America, and Japan. Recently the director of Japan’s Maritime Self-Defense Force Sasebo Historical Museum reported that the museum had preserved more than 3,000 artifacts with the method and that all remain in good condition.

## European Child Health Plan Adopted

In June 2004, the Fourth Ministerial Conference on Environment and Health adopted the Children’s Environment and Health Action Plan for Europe. The plan includes regional priority goals for ensuring children’s access to safe drinking water and adequate sanitation, reducing accidental deaths and injuries, promoting child-friendly urban planning, reducing respiratory disease, and reducing exposure to hazardous chemicals. The plan calls on the represented nations to implement national children’s environmental health plans by 2007. The meeting also sponsored a young journalists workshop and a youth parliament as part of the European Commission’s efforts to enlist young people as environmental stakeholders.

## A Vaccine for Medical Waste Burning

The NGO Health Care Without Harm has teamed with the Philippines Department of Health to prove large-scale immunizations can be conducted without the burning of medical waste. Incineration of syringes, needles, and other medical waste releases toxicants such as dioxin, mercury, and lead into the air. During the program, conducted during February 2004, about 18 million Filipino children were vaccinated for measles using an estimated 19.5 million syringes. The used syringes were collected in safety boxes and treated in either autoclave or microwave facilities, then buried in waste pits or put in concrete vaults. In 1999 the Philippines was the first nation to ban the burning of all waste, including medical waste.

## Figures and Tables

**Figure:**
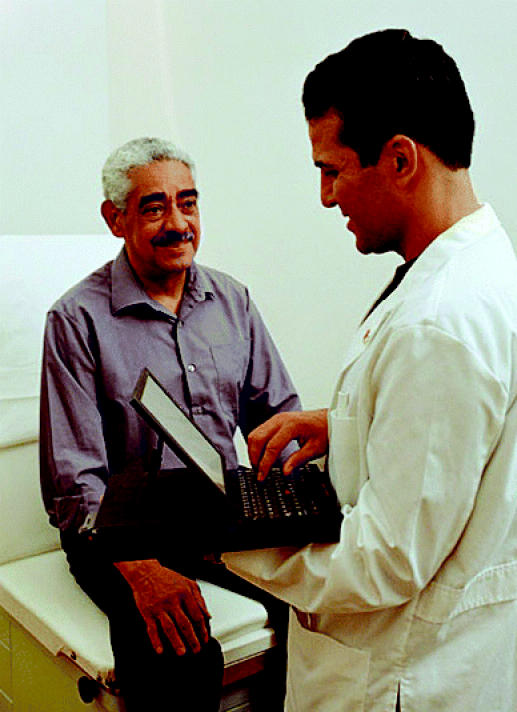


**Figure:**
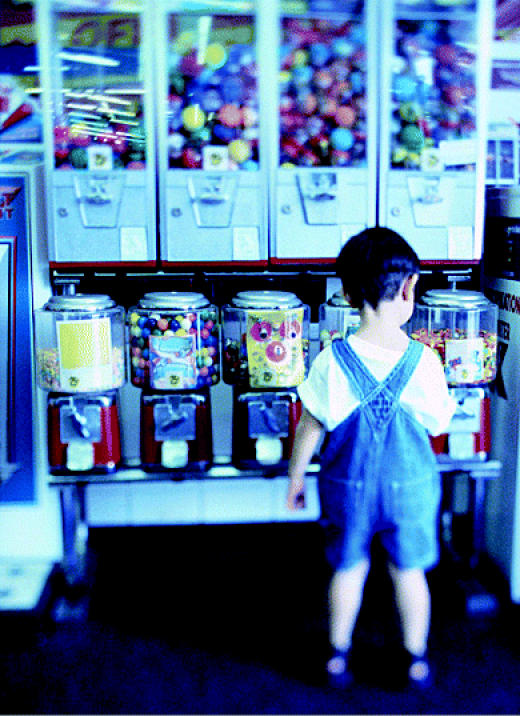


**Figure:**
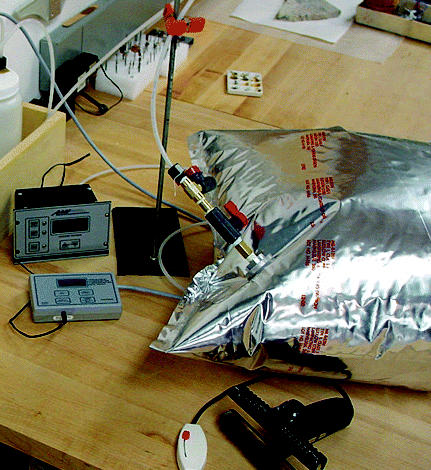


**Figure:**